# Systemic Resistance Induction of Potato and Tobacco Plants against *Potato Virus Y* by *Klebsiella oxytoca*

**DOI:** 10.3390/life12101521

**Published:** 2022-09-29

**Authors:** Mohsen Mohamed Elsharkawy, Fatimah O. Alotibi, Abdulaziz A. Al-Askar, Muhammad Adnan, Muhammad Kamran, Ahmed Abdelkhalek, Said I. Behiry, Muhammad Hamzah Saleem, Abdelmonim Ali Ahmad, Amr Ahmed Khedr

**Affiliations:** 1 Department of Agricultural Botany, Faculty of Agriculture, Kafrelsheikh University, Kafr El-Sheikh 33516, Egypt; 2 Department of Botany and Microbiology, College of Science, King Saud University, P.O. Box 2455, Riyadh 11451, Saudi Arabia; 3 Department of Plant, Soil and Microbial Sciences, Michigan State University, East Lansing, MI 48824, USA; 4 School of Agriculture, Food and Wine, The University of Adelaide, Adelaide, SA 5005, Australia; 5 Plant Protection and Biomolecular Diagnosis Department, ALCRI, City of Scientific Research and Technological Applications, New Borg El Arab City, Alexandria 21934, Egypt; 6 Agricultural Botany Department, Faculty of Agriculture (Saba Basha), Alexandria University, Alexandria 21531, Egypt; 7 MOA Key Laboratory of Crop Ecophysiology and Farming System Core in the Middle Reaches of the Yangtze River, College of Plant Science and Technology, Huazhong Agricultural University, Wuhan 430070, China; 8 Department of Plant Pathology, Faculty of Agriculture, Minia University, El-Minia 61519, Egypt

**Keywords:** *Klebsiella oxytoca*, biochar, PVY, jasmonic acid, salicylic acid, induced systemic resistance (ISR)

## Abstract

**Simple Summary:**

*Klebsiella oxytoca*, as a type of plant growth-promoting rhizobacteria (PGPR), was studied with regards to promoting plant growth and inducing plant systemic resistance against *Potato Virus Y* (PVY). The results of greenhouse experiments with tobacco and potato plants demonstrated that treatments with the *Klebsiella oxytoca* and biochar significantly enhanced the growth, while clearly lowering the disease severity and concentration of PVY. An RT-PCR analysis of the defense genes in the tobacco and potato treated with the *Klebsiella oxytoca* and biochar revealed an association with enhancing the systemic resistance of tobacco and potato to PVY. *Klebsiella oxytoca* and biochar may be considered valuable options to control PVY in potato and other Solanaceae crops.

**Abstract:**

*Potato Virus Y* (PVY) is a serious potato disease that may significantly decrease potato production. To suppress potato virus infection, several measures have been undertaken. The utilization of plant growth-promoting rhizobacteria is one of these methods. Biochar soil treatment is believed to provide plants with a number of advantages, including increased plant growth and the development of systemic resistance to a variety of plant diseases. The goal of this research was to see whether adding biochar and *Klebsiella oxytoca* to the soil might cause PVY resistance and enhance the involved mechanisms in PVY resistance. Potato and tobacco seedlings treated with *Klebsiella oxytoca* and biochar exhibited the same impact of significant symptom reduction, with complete negative ELISA findings, supporting the antiviral activity of *K. oxytoca* and biochar. Furthermore, owing to the connection between the ISR implicated substrates, significant amounts of polyphenol oxidase, catalase, and superoxide dismutase were observed in treated plants, with the same behavior as defense genes expression levels. It may be a step forward in the development of biochar and *K. oxytoca* as potential environmentally friendly disease control strategies against PVY.

## 1. Introduction

The potato (*Solanum tuberosum* L.) is the most frequently planted tuber crop in the world and the fourth biggest food crop overall behind rice, wheat, and maize, in terms of fresh production [1]. Each year, Egypt produces over 5 million tons of potatoes. However, the entire quantity exported reached roughly 755 thousand tons, which is about 13.5% of the total world production [1]. The most commercially significant virus of potato crops is *Potato Virus Y* (PVY), which is a member of the Potyvirus genus (Potyviridae family) that negatively impacts tuber production and quality, resulting in losses of 10–90%, according to the year, cultivar, and region [2]. Solanaceae plants, including peppers, tomatoes, and tobacco, are susceptible to PVY infection [3]. Over forty different types of aphids are responsible for insect transmission of PVY [4]. Chemical control of intracellular pathogens such as viruses is limited. Hence, in the case of PVY outbreaks, plants infected with the virus are destroyed, or pesticides are used to minimize the number of insects that serve as vectors for the pathogen [5]. To reduce the environmental danger posed by the use of chemical pesticides to control plant diseases, the need to explore biological control alternatives has increased.

PGPR (Plant growth-promoting rhizobacteria) have been identified as promising candidates among biological control agents, due to their ability to stimulate plant growth, improve quality, and prevent or reduce the spread of disease [6]. *Pseudomonas*, *Klebsiella*, *Enterobacter*, *Azospirillum*, *Burkholderia*, *Bacillus*, and *Serratia* are all examples of PGPR that stimulate plant development and suppress diseases [7,8]. *Klebsiella pneumoniae* HR1 improves germination percentage, shoot and root length, and shoot and root weight in *Vigna mungo* L. while decreasing the incidence of root rot caused by *Macrophomina phaseolina* [9]. The nitrogen-fixing bacteria *K. pneumoniae* NG14 colonizes the rice root surface [10]. *Klebsiella jilinsis* 2N3 enhances maize development and increases resistance to *Setosphaeria turcica*, which causes corn leaf blight [11]. Overall, PGPR isolates offer good prospects as future biological control agents for a wide range of plant diseases.

Every plant has defense mechanisms against diseases. A lack of an adequate defensive response may be the main reason why infected cells invade and alter the structure and function of surrounding cells, resulting in disease symptoms. Multiple variables, such as plant age and tissue type, influence the nature and effectiveness of a plant’s defenses against viruses. Plants have been shown to use a number of active and passive defensive mechanisms when attacked with diseases such as viruses. Induced resistance is a widespread mechanism throughout the plant, rather than confined to the diseased parts [12]. In order to reduce viral disease, plants’ natural defensive mechanisms, such as systemic resistance, might be induced via biotic or abiotic elicitors [13]. The defensive mechanism of the plant that is triggered by PGPR, such as *Klebsiella*, *Pseudomonas*, and *Bacillus*, prevents infection and does not lead to significant injury and death to the root system. For the identification of such plant–pathogen interactions, physiological, histological, and molecular investigations might be employed [14,15]. Different responses seen in the induced defense of susceptible and resistant plants show that successful resistance is dependent on the pathogenic process’s rate of progression [16]. Systemic resistance to plant diseases, as well as to some chemical elicitors, may be measured by monitoring the expression of the gene for pathogenesis-related protein 1 (PR1). PR1 proteins are ubiquitous in both monocotyledons and dicotyledons, demonstrating their significance in plant stress responses [17]. Additional viral infection may be prevented by the synthesis of PR proteins in uninfected areas of diseased plants, leading to a reduction in virus movement and replication [18,19]. Soybean growth was stimulated by *Klebsiella pneumoniae* SnebYK and regulated systemic resistance via the production of *PR1*, *PR2*, *PR5*, and *PDF1.2*, leading to reducing the invasion and development of *Heterodera glycines* [20].

The aim of this study was to compare the bio-elicitor performance of *Klebsiella oxytoca* and biochar against PVY^NTN^ infection in a greenhouse environment. Additional objectives include exploring the involved mechanisms of resistance through defense enzymes and the expression of induced resistance marker genes.

## 2. Materials and Methods

### 2.1. Isolation of the Rhizosphere Bacteria

To isolate growth-promoting bacteria, we collected soil samples from several sampling sites in Egypt. Each soil sample (1 g) was shaken for 10 min after being suspended in 9 mL of sterile distilled water (SDW). The solution was successively diluted to 10^−7^. On tryptic soy agar (TSA) plates, the diluted soil was plated in a 100 µL aliquot. For 1–3 days, plates were incubated at 32 °C. After being isolated, a single colony was cultured in tryptic soy broth at 32 °C for 1–2 days while being constantly shaken at 150 rpm, and it was then kept at −80 °C in 30% glycerol until use.

### 2.2. Identification of the Rhizosphere Bacteria

Genomic DNA was isolated using the Bacterial Genomic DNA Purification Kit (Thermo Scientific, Waltham, MA, USA), in accordance with the instructions provided by the manufacturer. The 16S rRNA gene sequence analysis was used to identify the bacterial strain. The 16S rRNA gene was amplified using PCR, followed by sequencing [21]. In BioEdit 5.0.9.1 and ClustalW, gene sequences were aligned and compared to the GenBank database. MEGA version 11.0.1 was used to create a phylogenetic tree using the neighbor-joining approach [22].

### 2.3. Effect of Klebsiella Oxytoca and Biochar on PVY^NTN^ Disease Severity

Infected potato plants were utilized to propagate PVY^NTN^, which was isolated in our previous study [23]. Potato tubers cv Spunta was given by the Agic. Res. Cen., Egypt. The potato tubers for the Spunta variety and tobacco seeds (*Nicotiana tabacum*) were grown in plastic pots (25 cm in diameter) filled with 3 kg of clay soil. Biochar was mixed with soil (at 1.5% *w*/*w*) before the planting of the biochar treatment group. Two weeks after planting, potato and tobacco plants were treated with 10 mL of cell suspension of *Klebsiella oxytoca* (CFU 10^−7^) 4 days before virus inoculation. PVY^NTN^ was mechanically inoculated on potato and tobacco leaves [23]. The first treatment included potato or tobacco plants mechanically inoculated with PVY^NTN^. The second treatment included potato or tobacco plants treated with 10 mL of *Klebsiella oxytoca* (CFU 10^−7^). The third treatment included potato or tobacco plants treated with biochar (1.5% *w*/*w*) before planting. All plants were kept in a pest-free greenhouse, with temperatures ranging from 28 °C during the day to 16 °C at night and a relative humidity of 70%. All plants were monitored on a daily basis, and any symptom of the disease was noted as it appeared. PVY^NTN^ development was assessed visually, and the percentage of infected plants was recorded based on when the first visible symptoms appeared on the plants. The whole experiment was repeated three times. Disease scale (0–10) was recorded [1] as follows: 0 = no symptoms; 2 = mild mosaic; 4 = mild necrosis; 6 = mild mosaic with mild necrosis; 8 = severe mosaic with necrosis; 10 = severe apical necrosis. The DS values were determined at 7, 14, and 21 days post-viral inoculation [24].

$$\mathrm{DS}=\frac{\Sigma \mathrm{disease}\;\mathrm{grade}\;\times \;\mathrm{number}\;\mathrm{of}\;\mathrm{plants}\;\mathrm{in}\;\mathrm{each}\;\mathrm{grade}}{\mathrm{Total}\;\mathrm{number}\;\mathrm{of}\;\mathrm{plants}\;\times \;\mathrm{highest}\;\mathrm{disease}\;\mathrm{grade}\;}$$

The area under disease progress curve (AUDPC) was recorded [1]. Potato or tobacco leaves from three separate plants were collected to ensure statistical reliability when measuring enzyme activity and RNA extraction across treatments. The fresh and dry weights of the shoots were recorded for each treatment (g).

### 2.4. Assessment of PVY^NTN^ Concentration by DAS–ELISA

After 3 WAI (weeks after inoculation), the virus concentration in potato and tobacco leaves was measured using a double-antibody sandwich enzyme-linked immunosorbent assay (DAS-ELISA) to detect PVY^NTN^ infection [1]. Triplicate DAS-ELISA tests were performed.

### 2.5. Assessment of Antioxidant Enzymes

The supernatant from leaf samples that had been homogenized in phosphate buffer pH 7 and centrifuged at 10,000× *g* was then employed in the subsequent tests of enzyme activity.

#### 2.5.1. Polyphenol Oxidase (PPO)

PPO activity was determined according to the method of Cho and Ahn [25]. For 10 min at room temperature, 500 µL of enzyme extract was mixed with 1 mL of Tris-HCl and 50 mM of quinone (pH 6). All readings were taken three times at 420 nm, and results are provided in units of µmol/g fresh weight.

#### 2.5.2. Catalase (CAT) Activity

The rate of H_2_O_2_ degradation at 240 nm was used to calculate CAT activity [26]. An enzyme extract (50 µL) was mixed with a potassium phosphate buffer solution (1 mL, 25 mM, pH 7) containing H_2_O_2_ (10 mM). CAT activity was expressed as µmol/g fresh weight.

#### 2.5.3. Superoxide Dismutase (SOD) Activity

The capacity of superoxide dismutase (SOD) to inhibit the photochemical reduction of nitro blue tetrazolium (NBT) was determined [23]. The mixture consisted of KH_2_PO_4_ (50 mM, pH 7.8), NBT (75 mM), L-methionine (10 mM), EDTA (0.1 mM), and riboflavin (20 mM) with enzyme extract (100 µL). After 15 min in the light, the tubes were incubated at 25 °C under two 15 W fluorescent lights. Finally, the absorbance at 560 nm was calculated. SOD activity was expressed as µmol/g FW.

### 2.6. Expression of PR-1b Gene by Real-Time RT-qPCR in Potato Plants

Total RNA was isolated from fresh potato leaves collected at 2 DAI (days after inoculation) for each treatment to analyze *PR-1b* gene expression. With a mortar, leaves were finely ground while submerged in liquid nitrogen. Total RNA was extracted from the powder by using an RNeasy Plant Mini Kit (QIAGEN, Hilden, Germany), and the resulting RNA was deposited in a different micro-centrifuge tube. High-capacity cDNA reverse transcription kits (Applied Biosystems, USA) were used to generate cDNAs, in accordance with the manufacturer’s instructions. Real-time PCR was used to amplify DNA. The *Coxa* gene was used as a housekeeping reference gene to standardize the expression levels of the other genes (Table 1). Melting curve analysis was used to confirm the identification and specificity of the RT-qPCR products. Biological treatment was performed in triplicate using the SYBR Green Mix and real-time equipment. The 2^−ΔΔCT^ technique was used to precisely quantify and determine the relative transcriptional level of each gene in the experiment [27].

### 2.7. Expression of Defense Genes in Tobacco by Reverse Transcription PCR

RNA isolation and cDNA synthesis from tobacco leaves of treated and control plants were performed as described above. RT-PCR amplification was performed on an aliquot of the obtained cDNA, as described by Elsharkawy et al. [30], to evaluate the transcription of a group of well-differentiated defense-related genes. The gene-specific primers used in these experiments, *PR-1a*, *Coi1,* and *Actin,* are listed in Table 2.

### 2.8. Statistical Analysis

The statistical software SPSS (version 21.0 for Windows; SPSS, IBM Corp., Armonk, NY, USA) was used for all analyses. The results were presented as means for each set of replicates, and any statistically significant differences were identified by one-way analysis of variance (ANOVA) and Tukey’s HSD test (*p* ≤ 0.05).

## 3. Results

### 3.1. Identification of Rhizosphere Bacteria

**Figure 1:**
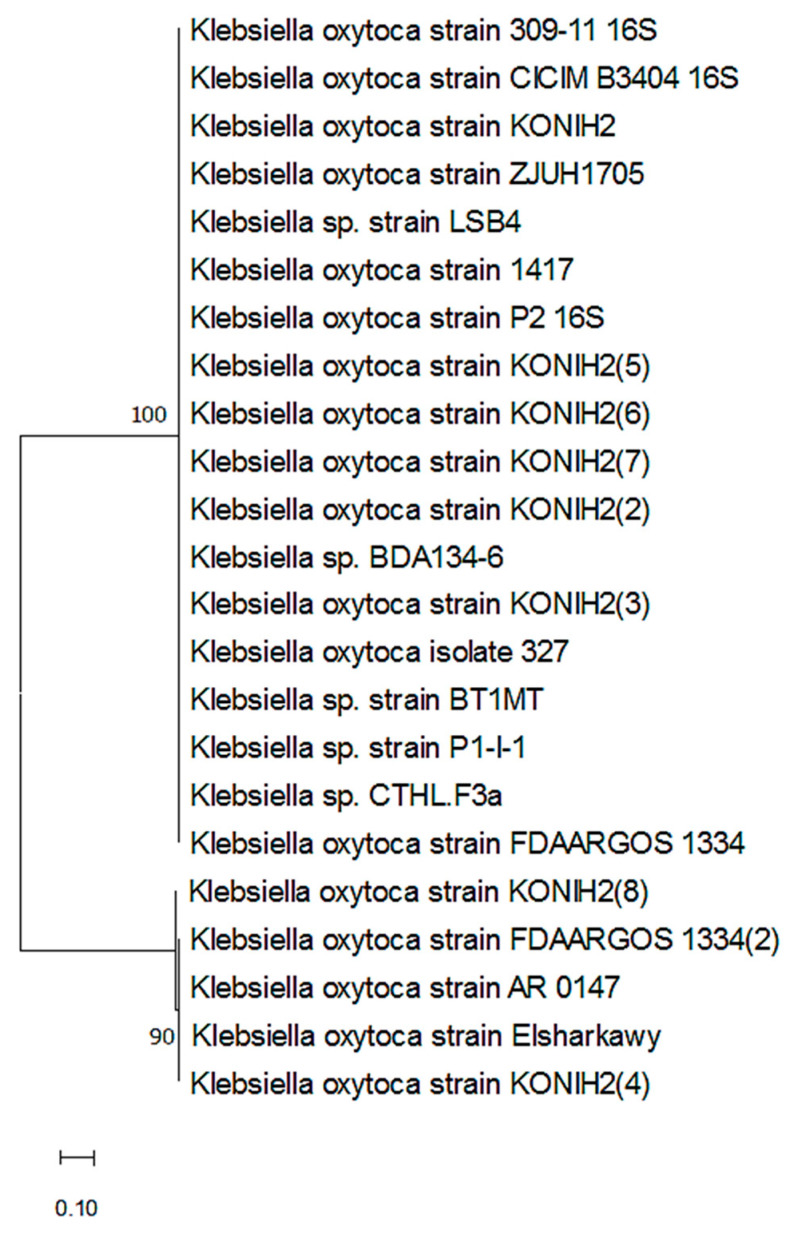
Phylogenetic dendrogram showing the position of the *Klebsiella oxytoca* strain Elsharkawy among phylogenetic neighbors.

To determine which isolates were present, PCR amplicons were used. The resulting phylogenetic tree from 16S rRNA sequences is shown in Figure 1. *Klebsiella oxytoca* was shown to be the most often detected bacterium when a nucleotide database blasting approach was used. Accession OP268604 denotes the submission of the partial 16S rRNA gene sequences for *Klebsiella oxytoca* strain Elsharkawy to NCBI. Figure 1 shows the phylogenetic relationships between selected isolate sequences and the isolates in the GenBank database, with 100% similarity with the *Klebsiella oxytoca* strain KONH2.

### 3.2. Effect of K. oxytoca and Biochar on PVY^NTN^ Disease Severity

**Figure 2:**
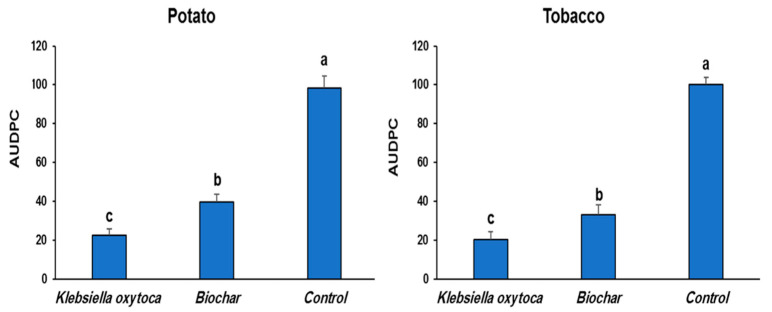
Influence of treatments with *Klebsiella oxytoca* and biochar on AUDPC of PVY^NTN^ in potato and tobacco. Values with the different letters are significantly different.

In comparison to untreated PVY^NTN^-infected control plants, potato and tobacco plants treated with *K. oxytoca* and biochar showed significantly reduced disease symptoms. The severity and appearance of symptoms in the plants were evaluated at 1, 2, and 3 WAI. Figure 2 shows that treatment with *K. oxytoca* was the most effective treatment, as measured by a reduction of apical necrosis symptoms on the potato plants. In the current research, using *K. oxytoca* resulted in a substantial decrease in disease severity for potato and tobacco, 77.3 and 59.9%, respectively, whereas biochar had a 79.9 and 66.9% reduction in disease severity in potato and tobacco, respectively.

### 3.3. Effect of Klebsiella oxytoca and Biochar on PVY^NTN^ Concentration

**Figure 3:**
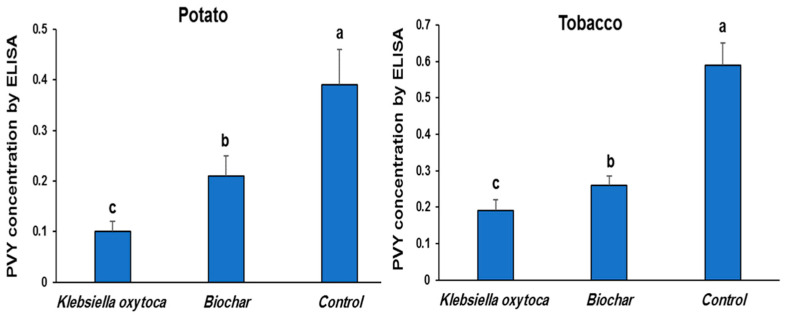
The effect of *K. oxytoca* and biochar on PVY^NTN^ concentrations in leaves of potato and tobacco plants using DAS- ELISA at 3 weeks after inoculation (WAI). Values with the different letters are significantly different.

According to the ELISA results, non-treated potato plants had the greatest viral accumulation level, whereas *K. oxytoca* and biochar plants had much lower viral accumulation levels (Figure 3). The protective efficacy of *K. oxytoca* against PVY infection was demonstrated by the considerable reduction in PVY accumulation levels in potato and tobacco leaves by 74.4 and 56.7%, respectively, compared to control plants.

### 3.4. Evaluation of the Effect of Treatment with Klebsiella oxytoca and Biochar on Potato Growth Parameters

Both *K. oxytoca* and biochar treatments had significant effects on the length and fresh and dry weights of the shoot systems. Based on the results of the statistical analysis, it is clear that all parameters were improved dramatically (Table 3). *K. oxytoca* displayed the highest shoot (15.7 cm), followed by biochar (11.5 cm). The fresh weights of potato shoots (7.8 and 5.9 g), were significantly increased by the *K. oxytoca* and biochar treatments, respectively (Table 3). Similarly, *K. oxytoca* potato plants’ shoot systems (0.81 g) had much higher dry weights than the biochar treatment (0.52 g) (Table 3).

### 3.5. Effect of Treatment with Klebsiella oxytoca and Biochar on Antioxidant Enzymes Activity

The results show that treating potato plants with *K. oxytoca* and biochar prior to viral inoculation alters their PPO, CAT, and SOD activities. Results show that, as compared to the control group, accumulated PPO was 0.21 and 0.09 µmol/g FW greater when subjected to *K. oxytoca* and biochar treatments, respectively (Table 4). The enzyme activity of CAT in potato plants treated with *K. oxytoca* at 4 days before inoculation with PVY was 0.56 µmol/g FW. However, CAT activity in potato plants treated with biochar was not significant compared with the un-inoculated control (0.44 and 0.45 µmol/g FW, respectively). No significant differences in SOD enzyme activity were observed between the *K. oxytoca* and biochar treatments (Table 4).

### 3.6. Effect of Klebsiella oxytoca and Biochar on the Expression of PR-1b in Potato

**Figure 4:**
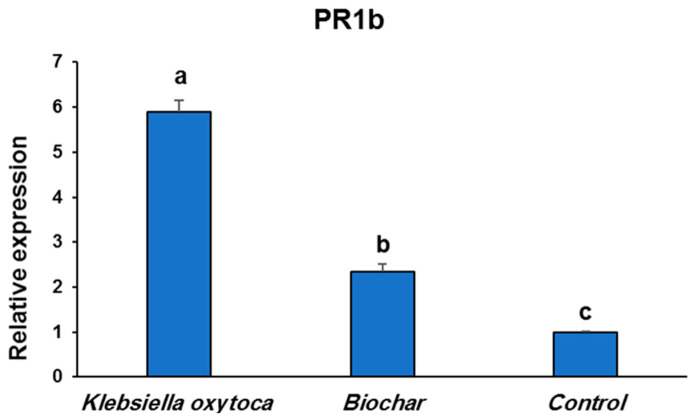
Expression of *PR1-b* gene in potato plants after application of *Klebsiella oxytoca* and biochar. Values with the different letters are significantly different.

Differential expressions of the *PR-1b* gene were observed between *K. oxytoca* and biochar-treated and control potato plants (PVY^NTN^-infected control). Relative expression levels of *PR-1b* were 5.9- and 2.4-fold, respectively, higher in the *K. oxytoca-* and biochar-treated potato plants, compared to the control (Figure 4). Therefore, *K. oxytoca* showed the highest fold change in *PR-1b* gene expression among all potato treatments, followed by biochar (Figure 4).

### 3.7. Effect of Klebsiella oxytoca and Biochar on the Expression of Pathogenesis-Related Genes in Tobacco

**Figure 5:**
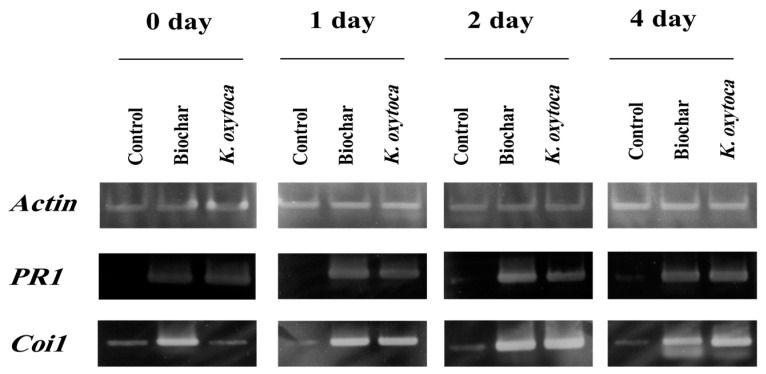
Expression of defense-related genes in leaves of tobacco plants treated with *Klebsiella oxytoca* and biochar before challenge inoculation with *Potato Virus Y*. *PR1* (SA-responsive) and *Coi1* (a regulator of the JA-dependent pathway) were analyzed by RT-PCR. *Actin*, which was expressed constantly, was employed as a control in RT-PCR.

The transcription of the *PR1* gene in tobacco plants began to be expressed one day after the induction treatments and lasted at high levels for four days post-inoculation in both *Klebsiella oxytoca* and biochar (Figure 5). In the *K. oxytoca* treatment, *Coi1* expression was first seen 1 day post-infection and was still increased at 4 days post infection. A peak in *Coi1* gene expression was seen 2 days post-inoculation in biochar, with a subsequent decrease at 4 dpi (Figure 5).

## 4. Discussion

Losses in agricultural output due to plant diseases, especially plant viral infections, are a major problem globally and threaten food security [33]. Economically and biologically, PVY is among the most devastating viruses. Depending on the host’s response, PVY variations may be separated into several strains, due to their wide range of serological, biological, and molecular characteristics [34]. The foliar symptoms caused by PVY^N^ and PVY^NW^ strains are scarcely evident, whereas the foliar symptoms caused by PVY^0^ and PVY^C^ strains are typically moderate and include mosaic lesions, leaf drop, crinkling, and dwarfing. On the other hand, PVY^NTN^ infection not only causes severe foliar symptoms but also leads to potato tuber necrotic ringspot disease, which has a devastating effect on tuber marketability. Since the 1980s, PVY^NTN^ has spread rapidly over potato crops in Europe and other continents, earning it the reputation as the most aggressive PVY strain [35]. An earlier infection of PVY^NTN^ was isolated in Egypt [36,37]. The PVY^NTN^ virus may infect almost all varieties of potatoes, leading to a wide range of symptoms that can range from severe necrotic ringspots on tubers and necrotic spots (local lesions) to wrinkles and mosaic chlorosis on leaves, new leaf deformation, and ultimately, plant death [38]. *Klebsiella oxytoca* and biochar were tested for their ability to induce resistance against the potato tuber necrotic strain (PVY^NTN^) in potato and tobacco. Significant decreases in PVY severity and accumulation were observed in potato and tobacco plants treated with *Klebsiella oxytoca* and biochar, compared with the control group. In comparison to control plants that had been infected with PVY and had severe symptoms of mosaic, including distorted leaves and apical necrosis, *Klebsiella oxytoca* treated plants were asymptomatic at 3 WAI. Similarly, *Streptomyces chibaensis* showed potential effects against banana bunchy top virus infection, and a significant reduction in symptoms was noticed when the treatment was given 10 days before viral inoculation [39]. A considerable suppressive effect of *K. oxytoca* against PVY^NTN^ was detected by detecting the virus concentration in potato leaves using DAS-ELISA. The protective efficacy of *K. oxytoca* and biochar against PVY infection was proven by a 74.4 and 56.7% reduction in PVY accumulation levels in potato and tobacco leaves, respectively compared to control plants. These results point to the possibility that *K. oxytoca* and biochar activate the host’s innate immune system and/or induce systemic resistance, which in turn inactivates and/or inhibits PVY replication. The findings are consistent with other studies that have shown positive effects from growth-promoting microorganisms on potato and tobacco plants [1,30]. It was reported that *Streptomyces olivaceus* might be used as a spray to effectively suppress cucumber mosaic virus infection [40,41]. In response to the PVY inoculation of potato, we observed considerable increases in three antioxidant enzymes: CAT, PPO, and SOD. Antioxidant enzyme PPO activity was two times higher in the *K. oxytoca* group (0.21 µmol/g FW) than in the biochar group (0.09 mol/g FW). PPO activity was considerably increased by biochar treatment by 55% (compared to the control). The ability of PPO to use a reactive oxygen species (ROS) as a substrate and produce lignin inside the cell wall acts as a physical barrier that can prevent the spread of disease [42]. However, *K. oxytoca* treatment showed a high increase in CAT activity levels (0.56 µmol/g FW) compared to the control and biochar treatment (0.45 and 0.44 µmol/g FW, respectively). CAT has been found to have a crucial role in safeguarding plant cells from ROS-induced oxidative damage after stress exposure [43]. Interestingly, SOD enzyme levels were significantly different in treated plants than from control treatments. There was no statistically significant difference in accumulation of SOD between treatments of *K. oxytoca* and biochar. SOD activity was considerably increased in all treatments’ leaf tissues, but it was much higher in the *K. oxytoca*-treated plants, compared to the control treatment. Defending against pathogen invasion is one of SOD’s primary roles by fortifying cell walls [44]. It is possible that the antioxidant enzyme activity shown in *K. oxytoca* and biochar treatments might be attributed to the wide variety of phenolic and flavonoid components. Otherwise, H_2_O_2_-induced oxidative stress may be reduced by increasing CAT expression, which activates the H_2_O_2_ signaling function in disease progression [45]. Therefore, application of *K. oxytoca* and biochar prior to PVY inoculation might be a viable technique for mitigating the negative consequences of viral infections. Our findings indicate that *K. oxytoca* and biochar improve the natural defenses and detoxification processes, leading to more rapid and effective responses to viral inoculation. Additionally, it is possible that the presence of antioxidant enzyme activities (CAT, PPO, and SOD) is essential for potato survival when subjected to viral stress. The application of *K. oxytoca* and biochar prior to PVY inoculation considerably protected potato plants from PVY by improving growth parameters, elevating the antioxidant system, and modulating gene expression. Therefore, this research may have generated a new PGPR–host–pathogen system by exploring the underlying mechanisms of PVY resistance. More research is needed later to understand all the alterations in the proteome of *K. oxytoca-* and biochar-primed potato.

It has been stated that the group of proteins called pathogen-related (PR) proteins is responsible for induced resistance and may effectively prevent the growth, multiplication, and/or spread of pathogens [46]. Two different types of PR genes (*PR1* and *Coi1*) were tested for their responses to PVY infection across *K. oxytoca* and biochar treatments in tobacco plants. Strong expressions of *PR1* (SA-responsive) and *Coi1* (a regulator of the JA-dependent pathway) were detected in tobacco plants. Similarly, the transcriptions of *PR1* and *Coi1* genes were significantly increased in tobacco plants treated with *Pencillium simplicissimum* in response to cucumber mosaic virus infection [30].

The PR genes such as *PR1* and *PR2* are particularly strong indicators of this systemic immune response [47]. *PR1b* levels were found to be significantly (*p* ≤ 0.05) higher in the treated potato plants and challenged with PVY, with a 2.4–5.9-fold higher change in relative expression level, compared to the control. For over twenty years, research has shown that salicylic acid and jasmonic acid play significant roles in plant immune activation and have established SA and JA as crucial plant signal phytohormone molecules [30,48]. Therefore, we believe that elicitor metabolite chemicals present in the *K. oxytoca* and biochar may activate JA and boost plant resistance to viral infection. The findings are consistent with previous research showing a considerable activation of *PR1* during viral infections of many plant species, including *Arabidopsis*, tobacco, potato, and tomato [30,49]. The stimulation of *PR1b* gene expression has protected against viral systemic infection in potato plants [46].

## 5. Conclusions

The PGPR strain *K. oxytoca* and biochar effectively suppressed the development of PVY, and the disease control efficacy was demonstrated to be primarily because of the activation of antioxidant enzymes and the expression of defense genes. This is the first study on the disease control efficacy of *K. oxytoca* and biochar on PVY. *K. oxytoca,* and biochar enhanced the expression of plant defense marker genes in both SA- and JA-signaling pathways. The results also exhibited the positive impact of *K. oxytoca* in potato growth and development. These findings indicate that *K. oxytoca* and biochar are potential biological control agents that suppress PVY via the induction of resistance.

## Figures and Tables

**Table 1:** Forward and reverse primers of potato used in RT-qPCR.

Primer	Forward	Reverse	Reference
*Coxa*	5-CGTCGCATTCCAGATTAT CAA-3	5-AA CTACGGATA TAT AAG AGC CAA AAC TG-3	[28]
*PR1b*	5-GTATGAATAATTCCACGTACCATATGTTC-3	5-GTGGAAACAAGAAGATGCAATACTTAGT-3	[29]

**Table 2:** Forward and reverse primers of tobacco used in RT-PCR.

Primer	Forward	Reverse	Reference
*PR1*	GTGTAGAACCTTTGACCTGGGA	TTCGCCTCTATAATTACCTGGA	[31]
*Coi1*	GGATTGACTGATTTGGCGAAGG	TCCCTCACTGGCTACAACTCGT	[31]
*Actin*	GGGTTTGCTGGAGATGATGCT	GCTTCGTCACCAACATATGCAT	[32]

**Table 3:** Effect of treatment with *Klebsiella oxytoca* and biochar on some vegetative growth parameters of potato.

Treatment	Shoot Length (cm/Plant)	Shoot Fresh Weight (g/Plant)	Shoot Dry Weight (g/Plant)
Control	8.4 c *	3.1 c	0.40 c
*Klebsiella oxytoca*	15.7 a	7.8 a	0.81 a
Biochar	11.5 b	5.9 b	0.52 b

* Values within the same vertical area with the same letter are not significantly different.

**Table 4:** Effect of *Klebsiella oxytoca* and biochar on the activity of the antioxidant enzymes of potato plants.

Treatment	PPO (µmol/g FW)	CAT (µmol/g FW)	SOD (µmol/g FW)
Control	0.04 c *	0.45 b	0.40 b
*Klebsiella oxytoca*	0.21 a	0.56 a	0.49 a
Biochar	0.09 b	0.44 b	0.50 a

* Values within the same vertical area with the same letter are not significantly different.

## Data Availability

Not applicable.
